# Dengue and the Heart: A Retrospective Study of Electrocardiographic Changes

**DOI:** 10.7759/cureus.93269

**Published:** 2025-09-26

**Authors:** Seetharaman M, Bhuvaneswari Kothendaraman

**Affiliations:** 1 General Medicine, Indira Gandhi Medical College and Research Institute, Pondicherry, IND

**Keywords:** arrhythmia, cardiac manifestations, conduction abnormalities, dengue, dengue fever, dengue hemorrhagic fever, dengue shock syndrome, ecg, sinus bradycardia, sinus node dysfunction

## Abstract

Introduction: Dengue fever, an arboviral infection, has been associated with various systemic complications, including cardiac involvement. Electrocardiographic (ECG) abnormalities in dengue are underreported but may indicate transient or serious myocardial dysfunction. This study aims to assess the prevalence and nature of ECG changes, especially sinus bradycardia, in patients with dengue fever and to examine their correlation with clinical and laboratory parameters.

Methods: A retrospective cross-sectional study was conducted at the Department of General Medicine, Indira Gandhi Medical College and Research Institute, Pondicherry, including 301 dengue-positive patients admitted between January 1, 2023, and December 31, 2023. Data were collected from medical records, including demographics, clinical features, comorbidities, laboratory values, and ECG findings.

Results: ECG abnormalities were found in 72 patients (23.9%). The most frequent finding was sinus bradycardia, observed in 40 patients (13.3%), followed by sinus tachycardia in 18 patients (6%) and ST-T wave changes in six patients (2%). Rare conduction abnormalities were also noted. No significant differences were observed in hematological, renal, or hepatic parameters between patients with and without bradycardia. Most patients with ECG changes were between 18 and 35 years of age. Gender distribution was nearly equal, and most had no significant comorbidities.

Conclusion: Sinus bradycardia is a notable ECG abnormality in dengue fever and may occur independently of laboratory or clinical severity markers. Routine ECG monitoring should be incorporated into dengue management protocols to identify cardiac involvement early and avoid complications.

## Introduction

Dengue is the most rapidly spreading mosquito-borne viral disease in the world, caused by the dengue virus, primarily transmitted by *Aedes aegypti* [1]. An estimated 50 million dengue infections occur annually worldwide, and 2.5 billion people live in dengue-endemic countries. The disease typically presents with fever, rash, headache, and myalgia. Some of the dengue patients may have increased capillary permeability and bleeding tendencies, but increasingly, cardiac involvement has been recognized as a complication [2,3]. Direct viral invasion or immune-mediated myocardial injury results in conduction abnormalities of the heart [4].

Electrocardiographic (ECG) changes in dengue patients may reflect autonomic dysfunction, myocarditis, or transient myocardial suppression [5-8]. Sinus bradycardia has emerged as a frequent but often overlooked finding, particularly in convalescent phases [4]. Myocardial involvement may range from mild, reversible changes to clinically significant arrhythmias and even myocarditis [5-8].

The objective of this study is to analyze the prevalence and pattern of ECG changes, particularly sinus bradycardia, in patients hospitalized with dengue and to explore any associations with clinical and laboratory parameters.

## Materials and methods

Study design

This was a retrospective, cross-sectional study conducted in the Department of General Medicine, Indira Gandhi Medical College and Research Institute, Puducherry, in July 2024.

Inclusion criteria

All dengue-positive patients aged 18 years or older who were admitted to the medicine ward between January 1, 2023, and December 31, 2023, were included.

Exclusion criteria

Patients with a history of pre-existing cardiac disease or those on medications known to affect heart rate were excluded.

Sample size

Based on a previous study by Poornima and John [4], which reported a prevalence of sinus bradycardia of 8.71% with a precision of 5% and a 95% confidence interval, the minimum required sample size was calculated as 127. After accounting for a 10% non-response rate, the minimum sample size was estimated at 140. In this study, 301 patients were included to improve generalizability. The sampling technique used was universal sampling.

Data collection

Case records of all eligible patients admitted during the study period were retrieved from the Medical Records Department. Demographic data, clinical presentation, co-morbidities, laboratory values, and ECG findings were systematically recorded using a structured data collection sheet.

Statistical analysis

Data analysis was performed using SPSS version 20.0 (IBM Corp., Armonk, NY, USA). Categorical variables were expressed as frequencies and percentages. Continuous variables were presented as mean ± standard deviation (SD) or median with interquartile range (IQR), depending on the distribution. The chi-square test or Fisher’s exact test was applied to assess associations between categorical variables, while the Student’s t-test or Mann-Whitney U-test was used for continuous variables. A p-value of <0.05 was considered statistically significant.

## Results

**Figure 1:**
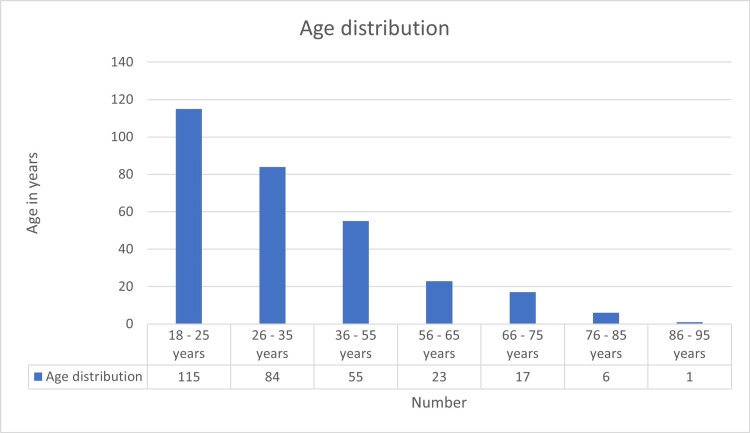
Distribution of dengue patients by age

A total of 301 patients were included in the study. The largest proportion belonged to the 18-25-year age group, accounting for 115 patients (38.2%), followed by 84 patients (27.9%) in the 26-35-year age group. Patients aged 36-55 years constituted 55 cases (18.3%). The 56-65-year-old and 66-75-year-old groups comprised 23 (7.6%) and 17 (5.6%) patients, respectively. Older age groups were less represented, with six patients (2.0%) in the 76-85-year group and a single patient (0.3%) in the 86-95-year group (Figure 1).

**Figure 2:**
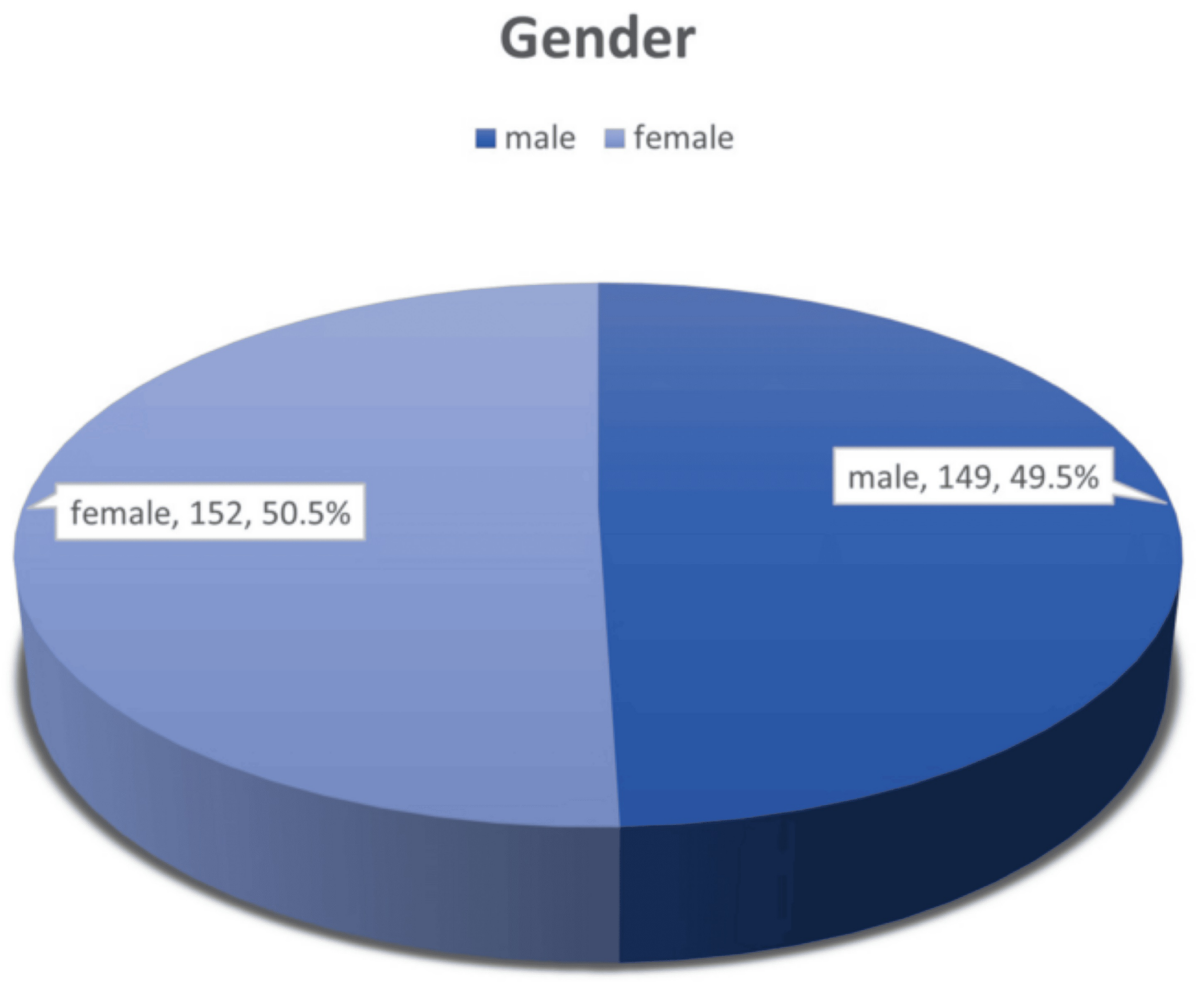
Distribution of dengue patients by gender

The gender distribution was nearly equal among the 301 participants, with female participants comprising 152 (50.5%) and male participants 149 (49.5%) (Figure 2).

**Figure 3:**
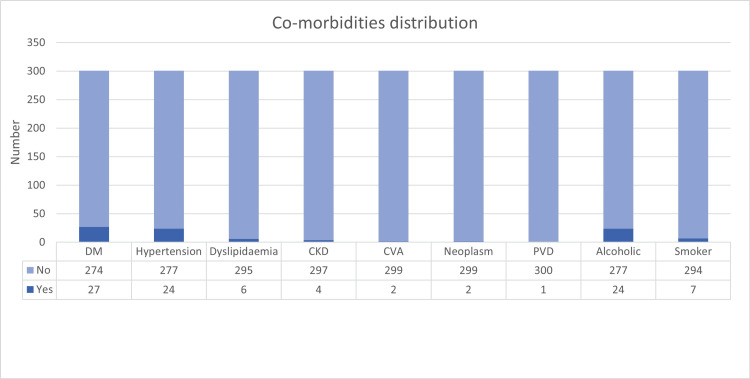
Distribution of co-morbidities among dengue patients DM: diabetes mellitus; CKD: chronic kidney disease; CVA: cerebrovascular accident; PVD: peripheral vascular disease.

Among the 301 patients studied, diabetes mellitus was present in 27 patients (9%) and hypertension in 24 patients (8%), making these the most common comorbid conditions. Dyslipidemia was reported in six patients (2%), while chronic kidney disease was observed in four patients (1.3%). Less frequently noted comorbidities included cerebrovascular accident in two patients (0.7%), neoplasm in two patients (0.7%), and peripheral vascular disease in one patient (0.3%). Regarding lifestyle factors, alcohol use was documented in 24 patients (8%) and smoking in seven patients (2.3%) (Figure 3).

**Figure 4:**
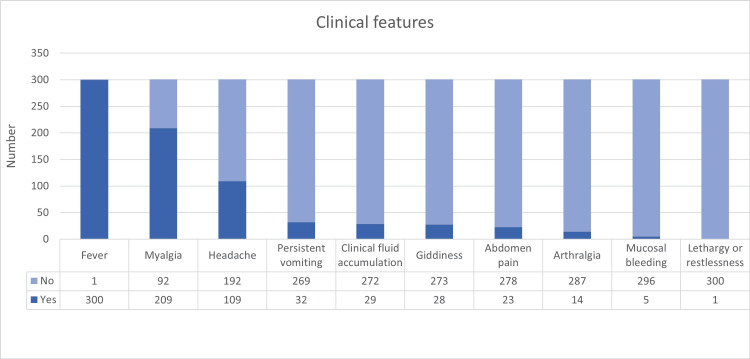
Distribution of clinical features among dengue patients

Fever was the most commonly reported symptom, observed in 300 patients (99.7%), followed by myalgia in 209 patients (69.4%). Other frequent symptoms included headache in 109 patients (36.2%), persistent vomiting in 32 patients (10.6%), clinical fluid accumulation in 29 patients (9.6%), and giddiness in 28 patients (9.3%). Less common manifestations were abdominal pain or tenderness in 23 patients (7.6%) and arthralgia in 14 patients (4.7%). Rare symptoms included mucosal bleeding in five patients (1.7%) and lethargy or restlessness in one patient (0.3%) (Figure 4).

**Figure 5:**
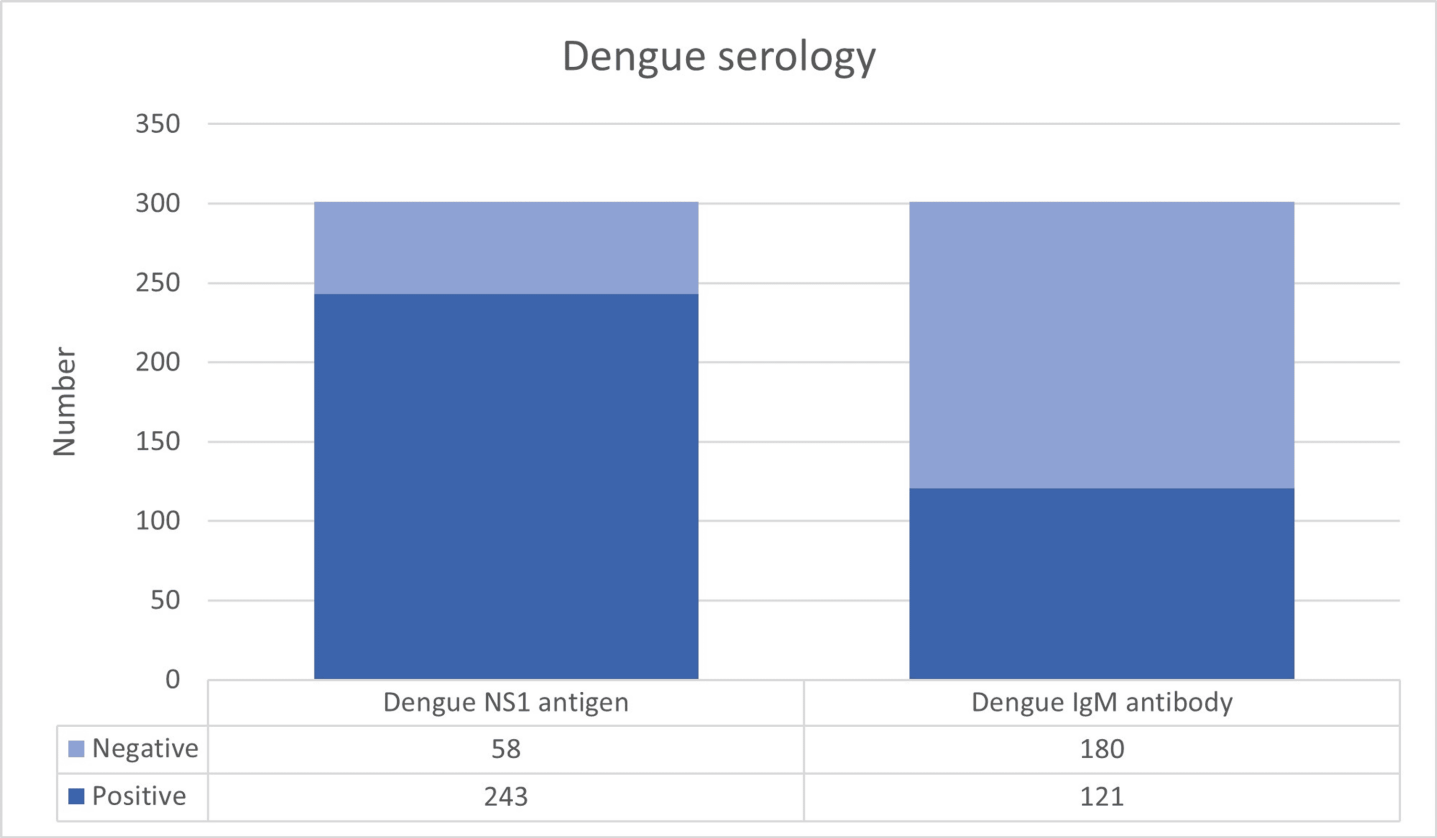
Diagnosis of dengue fever

Dengue NS1 antigen positivity was seen in 243 patients (80.7%), and IgM antibodies were positive in 121 patients (40.2%). These data suggest that laboratory confirmation of dengue was primarily achieved through NS1 antigen detection (Figure 5).

**Figure 6:**
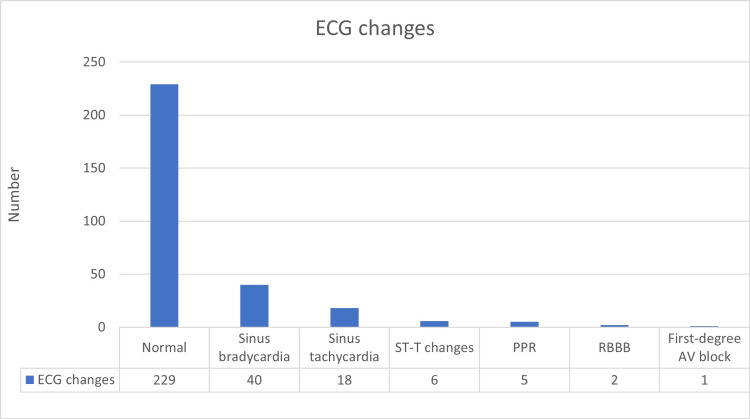
ECG findings among dengue patients PPR: poor progression of R wave; RBBB: right bundle branch block; AV: atrioventricular.

Analysis of ECG data among the 301 dengue patients demonstrated predominantly normal findings, observed in 229 patients (76.1%). Abnormal ECG findings were noted in 72 patients (23.9%). The most common abnormality was sinus bradycardia, present in 40 patients (13.3%), followed by sinus tachycardia in 18 patients (6%). ST-T wave changes, suggestive of ischemic or inflammatory changes, were observed in six patients (2%), while poor R-wave progression, indicative of possible ventricular conduction disturbances, was noted in five patients (1.7%). Right bundle branch block was infrequent, occurring in two patients (0.67%), and first-degree atrioventricular block was detected in only one patient (0.3%) (Figure 6).

The group analysis comparing patients with and without sinus bradycardia highlighted variations in laboratory parameters. Patients with sinus bradycardia had a marginally higher mean hemoglobin level (14.018 g/dL) compared to those without sinus bradycardia (13.452 g/dL). The mean packed cell volume (PCV) was also elevated in the sinus bradycardia group (40.793%) versus the non-sinus bradycardia group (38.776%). However, these differences were not statistically significant (p > 0.05).

Regarding total leukocyte count, the sinus bradycardia group had a higher mean count (5287.5 cells/µL) than the non-sinus bradycardia group (4813.41 cells/µL), but this difference was not statistically significant. Neutrophil counts were slightly elevated in the sinus bradycardia group (57.875%) compared to the non-sinus bradycardia group (54.387%), whereas lymphocyte and monocyte levels were marginally lower in the sinus bradycardia group. Platelet counts were slightly reduced in the sinus bradycardia group (61,525/µL) compared to the non-sinus bradycardia group (66,877.43/µL), although this finding also did not reach statistical significance (Table 1).

**Table 1:** Group statistics for complete blood count among dengue patients with and without sinus bradycardia cu mm: cubic millimeter; g: gram. A p-value of <0.05 was considered statistically significant.

Parameter	Sinus bradycardia	Frequency	Mean	Standard deviation	Standard error mean	Levene's test for equality of variances (F-value)	p-Value
Hemoglobin (g%)	Yes	40	14.018	2.3872	0.3775	1.064	0.303
	No	261	13.452	2.4105	0.1492		
Packed cell volume (%)	Yes	40	40.793	7.0448	1.1139	0.202	0.654
	No	261	38.776	6.3549	0.3934		
Total leucocyte count (cells/cu mm)	Yes	40	5287.500	2570.2626	406.3942	0.338	0.561
	No	261	4813.410	2542.7144	157.3901		
Neutrophil (%)	Yes	40	57.875	13.5499	2.1424	0.004	0.948
	No	261	54.387	13.6703	0.8462		
Eosinophil (%)	Yes	40	2.600	2.4683	0.3903	0.052	0.819
	No	261	2.935	2.7160	0.1681		
Basophil (%)	Yes	40	0.000	0.0000	0.0000	0.636	0.426
	No	261	0.199	3.1572	0.1954		
Lymphocyte (%)	Yes	40	34.650	11.9798	1.8942	0.255	0.614
	No	261	39.349	38.6065	2.3897		
Monocyte (%)	Yes	40	4.875	2.9105	0.4602	0.649	0.421
	No	261	5.536	3.9472	0.2443		
Platelet count (cells/cu mm)	Yes	40	61525.000	55509.2912	8776.7896	0.232	0.631
	No	261	66877.425	65122.3997	4030.9753		

The independent-samples test further corroborated the lack of significant differences across parameters such as hemoglobin, PCV, and leukocyte subtypes between the groups. These findings suggest that while subtle hematological variations may exist in patients with sinus bradycardia, they do not exhibit statistically robust differences when compared to patients without sinus bradycardia.

The group statistics for urea levels indicate that the mean urea level in patients with sinus bradycardia (19.975 ± 6.7120 mg/dL) was slightly lower than in those without sinus bradycardia (20.881 ± 11.0076 mg/dL). The independent-samples t-test revealed no statistically significant difference between the two groups (p = 0.613). Similarly, for creatinine levels, the mean value was marginally higher in patients with sinus bradycardia (0.905 ± 0.2650 mg/dL) compared to those without (0.879 ± 0.3752 mg/dL). However, this difference was not statistically significant (p = 0.672). The lack of significant differences suggests that urea and creatinine levels are not reliable markers of sinus bradycardia in dengue patients (Table 2).

**Table 2:** Group statistics for renal function test among dengue patients with and without sinus bradycardia mg/dL: milligrams per deciliter. A p-value of <0.05 was considered statistically significant.

Parameter	Sinus bradycardia	Frequency	Mean	Standard deviation	Standard error mean	Levene's test for equality of variances (F-value)	p-Value
Urea (mg/dL)	Yes	40	19.975	6.7120	1.0613	0.452	0.502
	No	261	20.881	11.0076	0.6814		
Creatinine (mg/dL)	Yes	40	0.905	0.2650	0.0419	0.016	0.900
	No	261	0.879	0.3752	0.0232		

The analysis of liver function parameters revealed comparable mean values for total bilirubin in patients with (0.643 ± 0.3226 mg/dL) and without sinus bradycardia (0.643 ± 0.3770 mg/dL), with no significant difference (p = 1.000). For direct bilirubin, the mean levels were slightly lower in patients with sinus bradycardia (0.250 ± 0.1301 mg/dL) than those without (0.268 ± 0.2412 mg/dL), though this was not statistically significant (p = 0.641). Indirect bilirubin levels were slightly higher in the sinus bradycardia group (0.400 ± 0.2342 mg/dL) compared to the non-dysfunction group (0.376 ± 0.1941 mg/dL), but this difference also lacked statistical significance (p = 0.477).

For liver enzymes, aspartate aminotransferase and alanine aminotransferase levels showed no significant differences between groups (p = 0.863 and p = 0.638, respectively). Similarly, alkaline phosphatase levels were higher in patients with sinus bradycardia (63.000 ± 49.4233 U/L) than in those without (56.184 ± 55.3163 U/L), but this was not statistically significant (p = 0.463). Overall, the findings suggest no strong association between liver function abnormalities and sinus bradycardia in dengue patients (Table 3).

**Table 3:** Group statistics for liver function test among dengue patients with and without sinus bradycardia mg/dL: milligrams per deciliter; AST: aspartate aminotransferase; ALT: alanine aminotransferase; ALP: alkaline phosphatase; IU/L: international units per liter. A p-value of <0.05 was considered statistically significant.

Parameter	Sinus bradycardia	Frequency	Mean	Standard deviation	Standard error mean	Levene's test for equality of variances (F-value)	p-Value
Total bilirubin (mg/dL)	Yes	40	0.643	0.3226	0.0510	0.093	0.760
	No	261	0.643	0.3770	0.0233		
Direct bilirubin (mg/dL)	Yes	40	0.250	0.1301	0.0206	1.287	0.257
	No	261	0.268	0.2412	0.0149		
Indirect bilirubin (mg/dL)	Yes	40	0.400	0.2342	0.0370	0.988	0.321
	No	261	0.376	0.1941	0.0120		
AST (IU/L)	Yes	40	98.250	78.5718	12.4233	1.828	0.177
	No	261	101.820	127.2169	7.8745		
ALT (IU/L)	Yes	40	84.500	70.8661	11.2049	0.104	0.747
	No	261	77.023	96.3555	5.9643		
ALP (IU/L)	Yes	40	63.000	49.4233	7.8145	0.009	0.924
	No	261	56.184	55.3163	3.4240		

## Discussion

ECG abnormalities are an increasingly recognized clinical finding in dengue infection, reflecting the systemic nature of the disease and its potential impact on the cardiovascular system. In our study, sinus bradycardia was the most frequently observed ECG abnormality, present in 40 patients (13.3%). This observation aligns with prior studies. Several studies have investigated the occurrence of sinus bradycardia and other ECG abnormalities in patients diagnosed with dengue fever (DF). An observational cross-sectional study conducted by Kumar et al. [9] involving 100 dengue patients reported sinus bradycardia in 23 cases (23%). A prospective study by Krishna et al. [10] at Nalanda Medical College and Hospital, Patna, evaluated 108 dengue patients and observed sinus bradycardia in 34 individuals (31.48%). A longitudinal study by Jadav et al. [11] conducted in South Gujarat, which included 104 dengue patients, identified abnormal ECG findings in 28 patients (26.92%), although the specific prevalence of bradycardia was not delineated. Similarly, a retrospective observational study by Poornima and John [4] in Kerala involving 341 dengue patients reported abnormal ECG changes in 72 patients, among whom sinus bradycardia was present in 30 cases (8.79%). In a study by Tarique et al. [12] in Pakistan, bradycardia was observed in 18 out of 116 dengue patients (15.52%). A cross-sectional study by Chatterjee et al. [13] in West Bengal involving 100 patients categorized 82 cases as DF and 18 as dengue hemorrhagic fever (DHF). Sinus tachycardia was noted in 28.04% of DF and 22.22% of DHF cases, while sinus bradycardia was reported in 10.97% of DF cases. A literature review by Parchani et al. [14] concluded that sinus bradycardia is the most common ECG abnormality observed in dengue patients. In a cross-sectional study by Papalkar et al. [15] with 60 dengue patients, sinus bradycardia was observed in nine patients (15%). In a study by Nerella et al. [16], focused on pediatric dengue patients, sinus bradycardia was reported in 29 children (19.3%). Gondhali and Bhattad [17] documented sinus bradycardia in 40 out of 110 dengue patients (36.4%). Similarly, Yadav and Kumar [18] reported a notably high incidence, with sinus bradycardia identified in 60 out of 100 dengue patients (60%). An observational comparative study by Tripathi et al. [19] evaluated 40 pediatric dengue patients, including 20 from the pediatric intensive care unit (PICU) and 20 from the general ward. Relative bradycardia was seen in 17 PICU patients (85%) compared to only two ward patients (10%), indicating a possible association with disease severity [19]. Finally, a cross-sectional study conducted in Patna by Talreja et al. [20] involving 70 dengue patients demonstrated sinus bradycardia in 32 cases (45.71%).

The underlying mechanisms of bradycardia in dengue are likely multifactorial. One proposed explanation is autonomic imbalance, particularly an increase in vagal tone, which is thought to occur in the setting of high fever and systemic viral illness. Additionally, dengue patients are prone to fluid shifts and electrolyte imbalances, such as hyponatremia or hypokalemia, especially during the critical phase of illness when plasma leakage is prominent. These imbalances may transiently impair cardiac conduction and contribute to the development of bradyarrhythmia. Another possibility is subclinical myocarditis [5-8].

An important finding of our study is the lack of significant association between sinus bradycardia and laboratory parameters such as complete blood count, liver function tests, and renal function tests [14-16]. This indicates that the occurrence of ECG abnormalities may be independent of the severity of laboratory derangements, which are often used to guide clinical assessment in dengue. Consequently, our results support the routine use of ECG monitoring in dengue patients, particularly during the critical and recovery phases, regardless of their laboratory profile. This approach may facilitate early identification of cardiac involvement and differentiation between benign and potentially serious arrhythmias, some of which may require more intensive monitoring or intervention.

Our findings related to age and gender distribution were also consistent with existing literature from India and Southeast Asia, where dengue predominantly affects young adults and adolescents. This trend is likely a reflection of regional endemicity and vector exposure patterns. Furthermore, we did not observe any sex-specific differences in ECG abnormalities, suggesting that the cardiovascular manifestations of dengue are not influenced by gender [2].

Limitations

The retrospective design, relatively modest sample size, and exclusion of pediatric patients may restrict the generalizability of the findings. Additionally, more sensitive diagnostic tools such as echocardiography or continuous Holter monitoring were not utilized, which might have provided further insights into transient or subclinical cardiac involvement. Future prospective studies with larger patient cohorts and the inclusion of advanced cardiac assessments are needed to better understand the full spectrum and clinical significance of cardiac manifestations in dengue infection.

## Conclusions

This study emphasizes that cardiac involvement, particularly sinus bradycardia, is a notable yet often underrecognized ECG abnormality in DF. This study also demonstrates that ECG abnormalities occurred predominantly in young adults, with an almost equal gender distribution and few notable comorbidities. Although these ECG changes are usually transient, their identification is clinically relevant for timely intervention and prevention of complications. By underscoring the need for vigilance, this work highlights the importance of integrating cardiovascular assessment into standard dengue care and encourages future research to clarify long-term cardiac outcomes.
